# Improved water use efficiency and shorter life cycle of *Nicotiana tabacum* due to modification of guard and vascular companion cells

**DOI:** 10.1038/s41598-018-22431-5

**Published:** 2018-03-12

**Authors:** Gabriela L. Müller, María V. Lara, Pablo Oitaven, Carlos S. Andreo, Verónica G. Maurino, María F. Drincovich

**Affiliations:** 1 Centro de Estudios Fotosintéticos y Bioquímicos (CEFOBI-CONICET), Facultad de Ciencias Bioquímicas y Farmacéuticas, Universidad Nacional de Rosario, Suipacha 531, 2000 Rosario, Argentina; 2 Institute of Developmental and Molecular Biology of Plants, Plant Molecular Physiology and Biotechnology Group, Cluster of Excellence on Plant Sciences (CEPLAS), Heinrich-Heine-Universität, Universitätsstraße 1, 40225 Düsseldorf, Germany

## Abstract

Severe droughts are predicted for the twenty-first century, which contrast with the increased demand for plant materials. Thus, to sustain future generations, a great challenge is to improve crop yield and water use efficiency (WUE), which is the carbon gained per water lost. Here, expression of maize NADP-malic enzyme (NADP-ME) in the guard and vascular companion cells of *Nicotiana tabacum* results in enhanced WUE, earlier flowering and shorter life cycle. Transgenic lines exhibit reduced stomatal aperture than wild-type (WT). Nevertheless, an increased net CO_2_ fixation rate is observed, which results in less water consumption and more biomass production per water used. Transgenic lines export sugars to the phloem at higher rate than WT, which leads to higher sugars levels in phloem exudates and veins. Leaf quantitative proteomic profiling revealed drastic differences in proteins related to cell cycle, flowering, hormone signaling and carbon metabolism between transgenic lines and WT. We propose that the increased sugar export from leaves in the transgenic lines alleviates sugar negative feedback on photosynthesis and thus, stomatal closure takes place without a penalty in CO_2_ assimilation rate. This results in improved WUE and accelerated overall life cycle, key traits for plant productivity in the near future world.

## Introduction

Severe and widespread droughts over many land areas are predicted for the twenty-first century due to global climate change^1,2^. These predictions seriously contrast the need to increase the food supply for a growing world’s human population^3^, anticipating an upcoming competition for soil and water resources between agricultural production and urban growth. To sustain future generations, a great challenge for our society is to extent arable surfaces and increase crop yield and water use efficiency (WUE), defined as the ratio of carbon gain (biomass production) per water transpired^4–6^. Current strategies to improve crop yield focus on the engineering of photosynthesis, such as the introduction of bypasses to photorespiration, the implementation of a C_4_ engine in C_3_ plants, the optimization of antenna size, or the acceleration of recovery from photoprotection^7–9^. Crassulacean acid metabolism (CAM), a specialized photosynthesis with enhanced WUE, has also emerged as a possible solution for future demand challenges of food and water resources^10,11^. Although highly innovative, many of these strategies are not expected to lead to agricultural applications in the near future, especially those that rely on the modification of highly complex plant traits. Moreover, in several cases, improved WUE by genetic manipulation leads to reduced photosynthesis, and vice-versa; and, thus, it is concluded that success in simultaneous improvement of photosynthesis and WUE may take longer than suggested^6^.

Stomatal pores, formed by a pair of guard cells in the leaf epidermis, are the main gates that control CO_2_ and water vapour exchange with the environment in land plants. Thus, stomatal movements are crucial for plant yield and WUE, and a fine-tuning of stomatal activity is needed to allow CO_2_ influx but also to avoid water loss when water is scarce. Stomatal movements are controlled by inorganic ions, such as K^+^, metabolites such as sucrose and malate, redox signals, and the phytohormone abscisic acid (ABA)^12,13^. A complex guard cell metabolism, as well as mesophyll-derived metabolites that act as signals, regulate the fine tuning of stomatal movements^14^. Among metabolites modulating guard cell movements, malate plays a crucial role^15–21^. During stomatal opening, concomitantly with K^+^ uptake, malate is imported from the apoplast, as well as synthesized in guard cells, e.g. from pyruvate and/or PEP. On the contrary, malate efflux from guard cells, and/or its metabolization, are important mechanisms during stomatal closure^14,16,19^. Besides, malate also modulates vacuolar chloride channels, which are also involved in stomatal opening^22^. Importantly, different results with mutant or transgenic lines have indicated that manipulation of stomatal movements have great impact on photosynthesis and WUE; however, the impact is generally opposite on these parameters^6^. In some cases, such as in the disruption of *Arabidopsis thaliana* plasma membrane malate transporters of guard cells (atquac1 knockout mutant), the drastic modifications in the dynamics of stomatal movements resulted in higher photosynthetic rates, altered respiration and higher biomass under well-watered conditions^17,21^.

Apart from the role of malate in stomatal movements, this C_4_ acid is involved in several biochemical processes, such as the supply of CO_2_ for carbon fixation during C_4_ or CAM photosynthesis, the production of reductive power for fatty acid synthesis, the regulation of the cellular pH status, and the exchange of reduced equivalents between cellular compartments^23^. Malate is also an important form of fixed carbon that can be rapidly metabolized during the night or under energy-demanding conditions^24,25^. During carbon assimilation, C_3_ plants store photosynthetic products in leaves not only in the form of sugars, but also as malate and other organic acids^26,27^. Modification of malate levels has dramatic effects on sugar metabolism, as it was demonstrated in the case of transitory starch metabolism^25,28^. Moreover, changes in carboxylic acid abundances, including malate, are perceived and signaled in *A*. *thaliana* inducing transcriptional modifications of numerous nuclear-encoded genes^29^.

Considering the key role of malate in cellular metabolism, and that the manipulation of stomatal function is a promising approach for the improvement of WUE and plant productivity, we integrated the plastidic non-photosynthetic NADP-malic enzyme from *Zea mays* (ZmnpNADP-ME)^30,31^ into the *Nicotiana tabacum* genome using the Potassium channel 1 (KAT1) promoter, which drives expression to both guard cells^32^ and companion cells of sieve elements^33^. The transgenic tobacco lines obtained show decreased stomatal aperture, enhanced phloem loading of sugars, and shorter flowering time; all traits that are highly correlated to plant productivity and efficient water usage.

## Results

### Expression of ZmnpNADP-ME in tobacco using the KAT1 promoter

**Figure 1:**
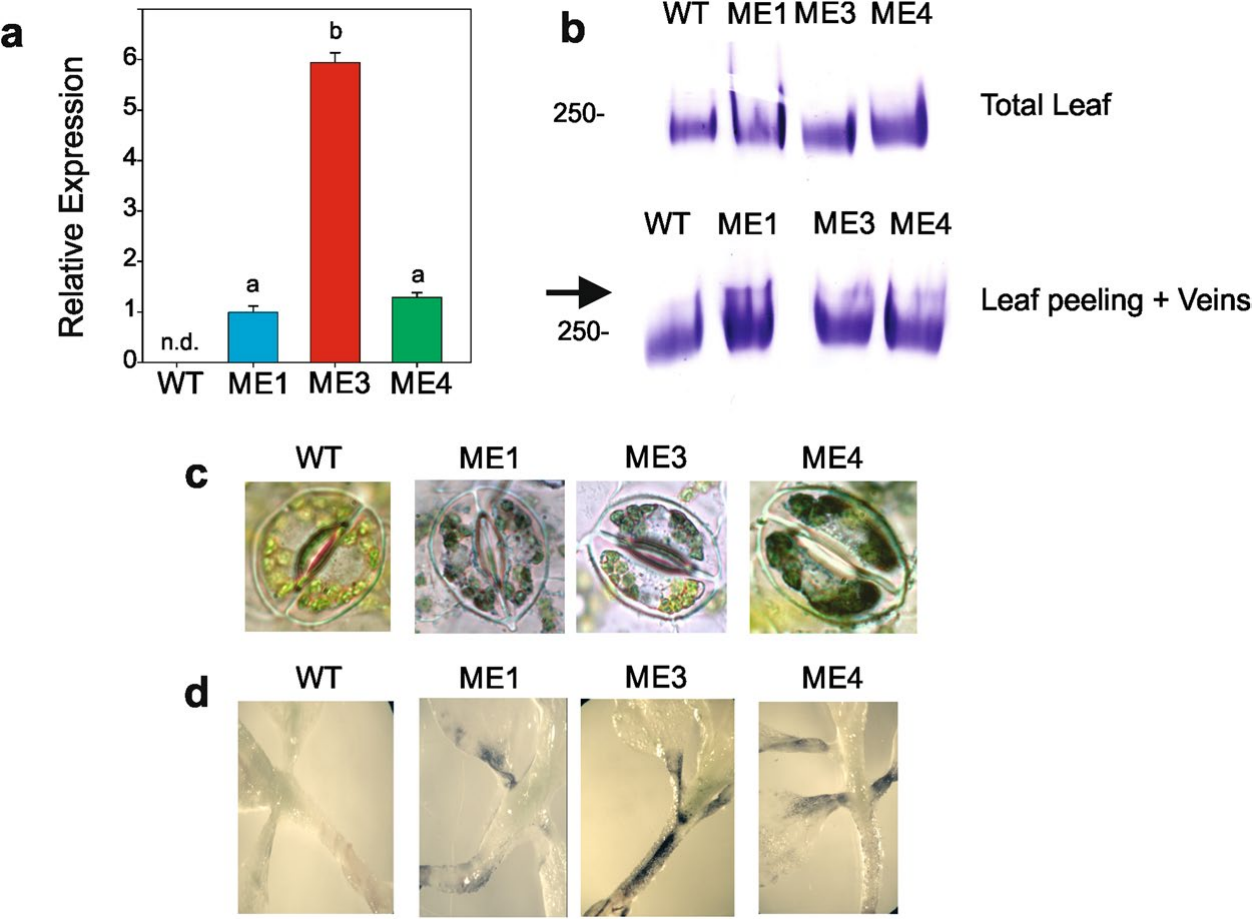
Transcript level and activity of ZmnpNADP-ME in *KAT1::ZmnpNADP-ME* lines. (**a**) Transcript levels of *ZmnpNADP-ME* in leaves of transgenic lines relative to the amount measured in ME1. Means of three independent measurements are shown. The bars indicate standard deviations. Values not labeled with an identical letter are significantly different (*P* < 0.05). *ZmnpNADP-ME* was not detected (n.d.) in WT. (**b**) NADP-ME activity assay after native PAGE of WT and transgenic lines. Approximately 10 mU of total NADP-ME activity from leaves (total leaf) and from epidermal strips (leaf peeling plus veins) were loaded in each well. The arrows point out the additional bands detected in transgenic lines in the lower panel. Full-length gel is included in Supplemental Fig. S2. (**c**) *In situ* NADP-ME activity assay in epidermal peels of 7 week-old WT and transgenic plants. Images of stomata were obtained by light microscopy. Activity was not detected when the assay was conducted in the absence of L-malate (not shown). (**d**) *In situ* NADP-ME activity assay in hypocotyls of 14 day-old seedlings WT and transgenic plants. Activity was not detected when the assay was conducted in the absence of L-malate (not shown).

The full-length sequence of *ZmnpNADP-ME* (Gene Bank: AY315822) fused to *Arabidopsis thaliana* Potassium channel 1 (KAT1, Gene Bank: U25088; At5g46240) was introduced in *N*. *tabacum*. Three tobacco *KAT1::ZmnpNADP-ME* transgenic homozygous lines (ME1, ME3, and ME4), obtained from independent transformation events, were selected for further analyses. *ZmnpNADP-ME* was expressed at different levels in the transgenic lines; while, as expected, *ZmnpNADP-ME* was undetectable in WT plants (Fig. 1a).

We analyzed NADP-ME activity in leaves of transgenic and WT plants by native gel electrophoresis. When using total leaf protein extracts, only one band, which molecular mass (250 kDa) corresponds to the native tobacco NADP-ME^34^, was detected in WT and transgenic plants (Fig. 1b, upper panel). However, when using protein extracts from leaf epidermal strips, which consisted in leaf peels with their associated veins, an additional band of lower mobility than the one corresponding to the native tobacco NADP-ME was detected only in the three transgenic lines, but not in WT (Fig. 1b, lower panel). The mobility of this additional band with NADP-ME activity corresponds to the mobility of ZmnpNADP-ME in maize organs^30^; so, we propose that the lack of detection of this band when using total protein extracts, may be due to the compartmentalized expression of the ZmnpNADP-ME.

We also assayed NADP-ME activity *in situ* using tobacco leaf epidermis peelings of adult plants and 14 days-old tobacco seedlings^35^. When using epidermal peelings, NADP-ME activity was detected in guard cell chloroplasts of the transgenic lines, and was not detected in WT (Fig. 1c). In the case of tobacco seedlings, NADP-ME activity was detected near the vasculature of stems and petioles of the transgenic lines, but not in WT (Fig. 1d).

The pattern of NADP-ME activity of the three *KAT1::ZmnpNADP-ME* transgenic tobacco lines, assayed both *in vivo* and by native gels of protein extracts, agrees with the reported expression of the KAT1 promoter in guard cells^32^ and phloem companion cells^33^.

### Phenotypic characterization of *KAT1::ZmnpNADP-ME* tobacco lines

**Figure 2:**
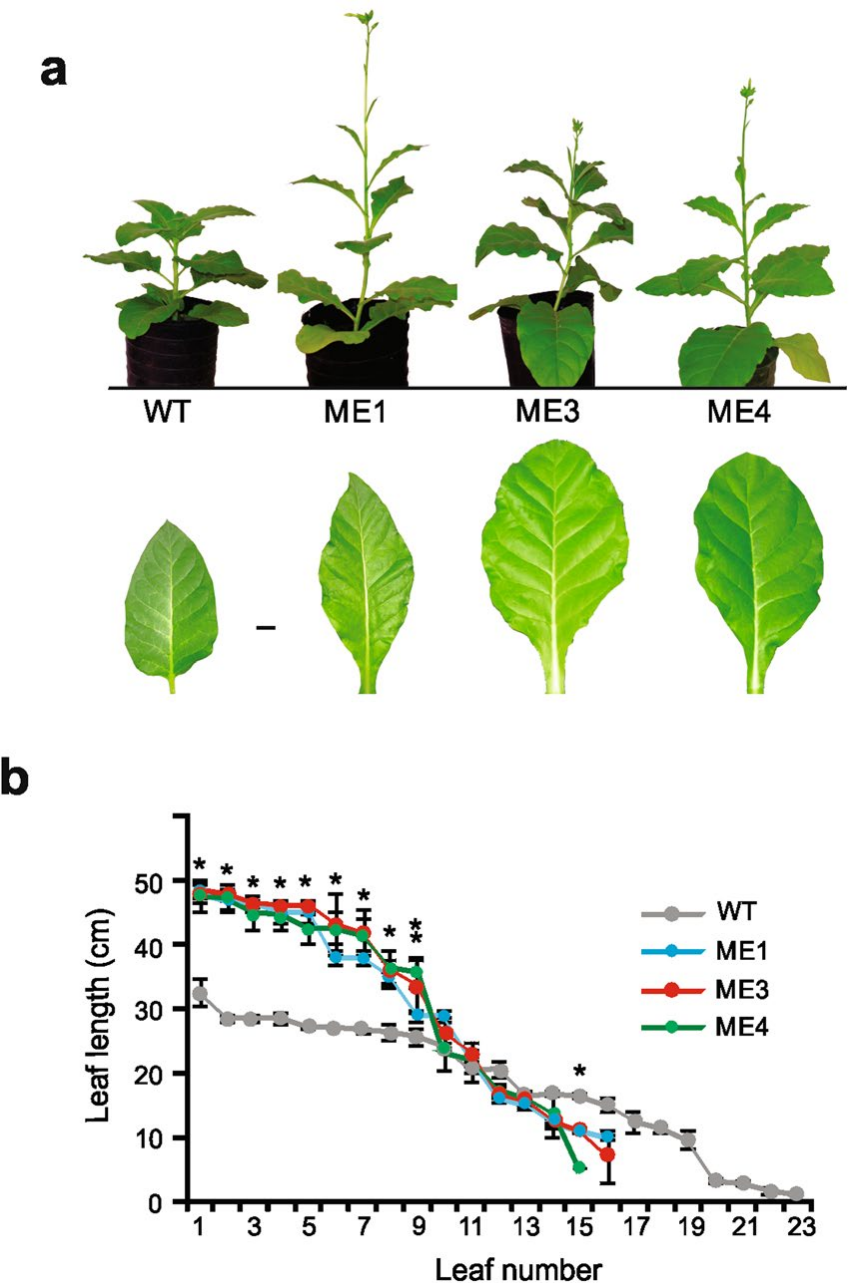
Phenotype of 10 week-old KAT1::ZmnpNADP-ME tobacco lines. (**a**) Comparative growth of 10-week old WT and transgenic plants under normal conditions (30/18 °C 12/12 h day/night period, 200 μmol m^−2^ s^−1^ of PPDF, 400 ppm CO_2_ and 90% FC). At the bottom, the third leaf from the base to the top of the WT and transgenic lines are shown. (**b**) Length of leaves of 10 week-old WT and transgenic lines. The leaves were numbered from the base to the top of each plant. The maximum length of each leaf was recorded. * indicates that length measured in ME1, ME3 and ME4 is statistically significantly different from the WT (*P* < 0.05). ** indicates that length measured only in ME4 is statistically significantly different from the WT (*P* < 0.05).

Ten week-old WT and transgenic lines were phenotypically characterized. At this growth stage, all *KAT1::ZmnpNADP-ME* lines exhibited increased height (110 cm on average) compared to the WT (66 cm on average; Table 1; Fig. 2a). Internode lengths were variable in the transgenic lines, from 3.6 cm on average at the base to 18 cm on average at the top of the plants (Table 1, Fig. 2a). On the contrary, WT plants exhibited uniform internode lengths (3.9 cm on average; Table 1, Fig. 2a).

**Table 1:** Phenotypic characterization of 10 week-old *KAT1::Zm NADP-ME* plants.

	WT	ME1	ME3	ME4
Plant height (cm)	66 ± 5^a^	118 ± 9^b^	110 ± 9^b^	102 ± 5^b^
Internode type Internode length (cm)	Constant 3.9 ± 0.5	Variable 3.3 ± 0.6 (at the base) 17 ± 6 (at the tip)	Variable 3.5 ± 0.4 (at the base) 18 ± 4 (at the tip)	Variable 4.1 ± 0.9 (at the base) 19 ± 7 (at the tip)
N° of leaves/plant	22.3 ± 0.6^a^	14.5 ± 1^b^	14.6 ± 0.5^b^	13.5 ± 0.6^b^
N° of flower shoots/plant	0	2–6	2–6	2–4

The results presented are an average (±SD) of the measurements performed on at least five plants belonging to each type. Values not labeled with an identical letter are statistically significantly different (p < 0.05).

The number and length of the leaves was also different when comparing the transgenic *KAT1::ZmnpNADP-ME* lines with the WT. The transgenic plants possessed fewer leaves than the WT; however, the first leaves from the base (leaf numbers 1 to 8) were significantly longer than those of the WT (Fig. 2a,b). The leaves of the transgenic plants also presented other distinct morphological characteristic: the leaf limb extended with the petiole until it joined the stem (Fig. 2a).

At ten weeks of growth, the presence of flower shoots was evident in the *KAT1::ZmnpNADP-ME* transgenic lines, which presented 2 to 6 flower shoots (Table 1; Fig. 2a). At this growth stage, the WT did not have any flower shoots. Thus, the flowering time (the time of plant growth until setting the first flower buds) was substantially shorter for the transgenic lines than for the WT (10 ± 1 vs. 15 ± 1 weeks). According to flowering time, the total life cycle (time until producing mature seeds) of the *KAT1::ZmnpNADP-ME* transgenic lines was completed in 16 ± 1 week while the WT needed 22 ± 2 weeks to complete the life cycle.

### Water consumption and biomass production of *KAT1::ZmnpNADP-ME* tobacco lines

After 4 weeks of growth of tobacco WT and *KAT1:ZmnpNADP-ME* lines, water added per day to assure 90% Field Capacity (FC) was recorded. The transgenic lines consumed significantly less water per day, between 71 to 89%, compared to the WT (Table 2). Total water consumed from 4 weeks of growth and until 11 weeks of growth was significantly lower in *KAT1:ZmnpNADP-ME* lines than in WT (Table 2).

**Table 2:** Duration of life cycle, water consumption and biomass production of *KAT1::Zm NADP-ME* plants.

	WT	ME1	ME3	ME4
Flowering time (weeks)	15 ± 1^a^	10 ± 1^b^	11 ± 1^b^	10 ± 1^b^
Life cycle completion (weeks)	22 ± 1^a^	16 ± 1^b^	17 ± 1^b^	16 ± 1^b^
Water per day (L)	0.080 ± 0.03^a^	0.071 ± 0.05^b^	0.068 ± 0.03^b^	0.057 ± 0.03^c^
Total water from 4 to 11 weeks of growth (L)	4.0 ± 05^a^	3.4 ± 0.3^b^	2.9 ± 0.3^c^	2.7 ± 0.2^c^
Aerial DW (g) (11 week-old)	10.0 ± 0.5^a^	9.8 ± 0.9^a^	10.4 ± 0.9^a^	8.9 ± 0.8^a^
Terrestrial DW (g) (11 week-old)	3.8 ± 0.5^a^	3.8 ± 0. 8^a^	3.6 ± 0. 7^a^	3.7 ± 0. 6^a^

Tobacco WT and *KAT1::ZmnpNADP-ME* lines were grown at 90% field capacity (FC). Flowering time and time in weeks required to complete total life cycle is indicated. After 4 weeks of growth, water added per day to assure 90% FC is indicated. Total water needed at 90% FC from 4 weeks until 11 weeks of growth was also recorded. Aerial (stems and leaves) and terrestrial (roots) dry weight (DW) of 11 week-old plants is also indicated. Values not labeled with an identical letter are statistically significantly different (p < 0.05).

We compared the biomass production of *KAT1::ZmnpNADP-ME* lines and WT at 11 weeks of growth. Aerial (leaves and stems) and terrestrial (roots) biomass was not significantly different when comparing transgenic and WT plants (Table 2). Considering that the *KAT1::ZmnpNADP-ME* transgenic lines consume significantly less water than WT (Table 2), they require less water than the WT to produce almost the same biomass.

### Stomatal aperture and CO_2_ fixation rate of *KAT1::ZmnpNADP-ME* tobacco lines

**Figure 3:**
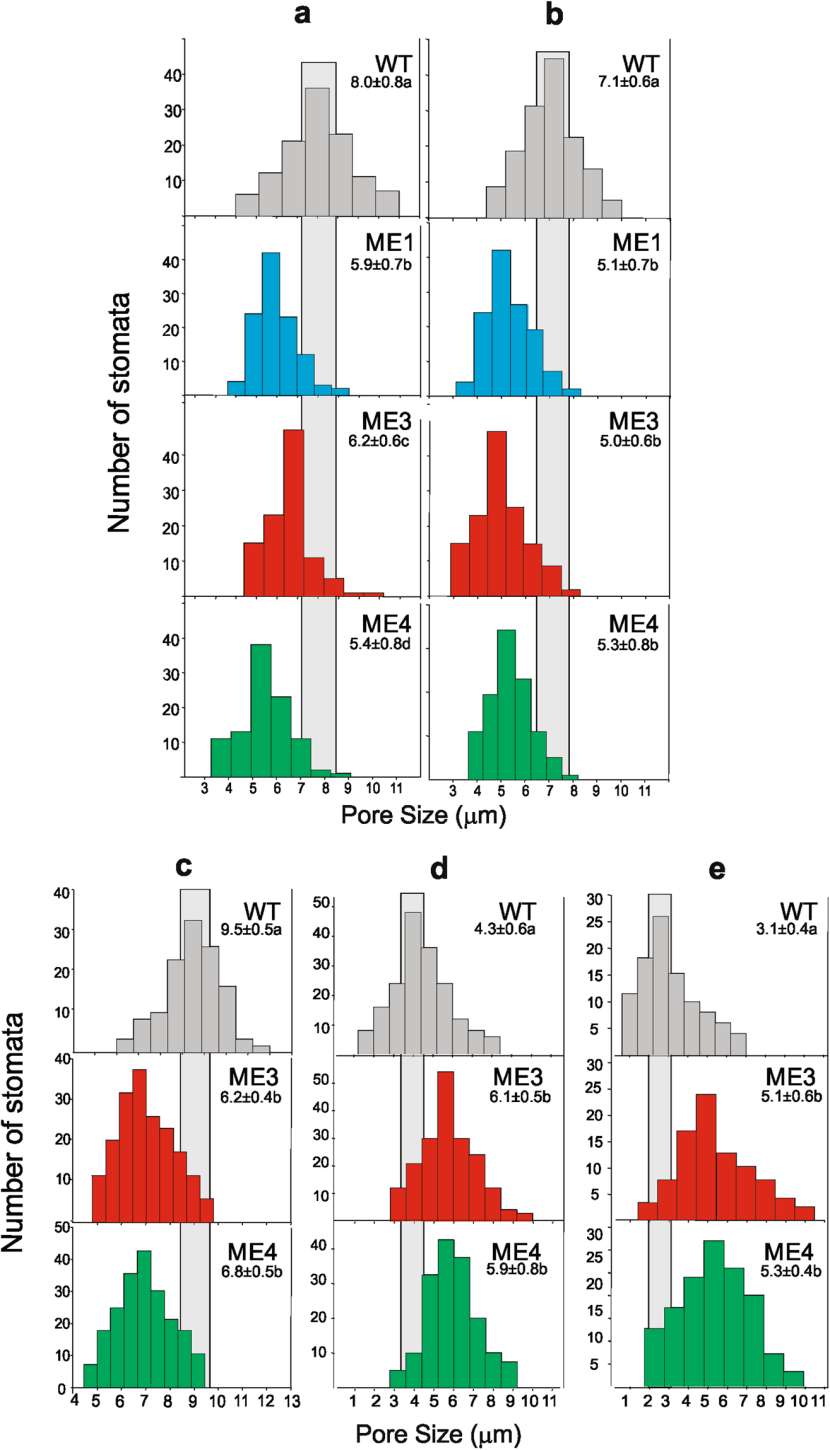
Stomatal pore size in *KAT1::ZmnpNADP-ME* plants. Histograms of stomatal pore size (stomatal aperture) of WT and transgenic plants at the beginning (morning, **a**) and at the end (evening, **b**) of the light period under 90% % FC and 400 ppm CO_2_. Modulation of stomatal size by acidification (**c**) or under high levels of CO_2_ (700 ppm, **d**; and 1200 ppm, **e**) was evaluated at the beginning of the photoperiod. Gray regions represent the 95%-confidence interval for the WT mean pore size. The mean of stomatal pore size (in μm) is indicated in each graph. For each condition, values not labeled with an identical letter differ significantly (*P* < 0.05).

To determine whether the changes in water consumption of transgenic lines were related to a differential stomatal behavior, the stomatal pore size was examined in leaf peels of 7 week-old plants at the beginning and at the end of the light period. The stomata of all transgenic lines had significantly reduced pore sizes compared to the WT, both at the beginning and at the end of the light period (*P* < 0.05; Fig. 3a,b). The size of stomata pores of the three transgenic lines was between 68 to 77% the size of the stomatal pores of WT (Fig. 3a,b).

The response of stomata to changes in the pH and in the CO_2_ concentration was investigated. Acidification of leaf peels due to addition of weak acid butyrate conducted to the opening of stomata in WT plants. In contrast, we observed no changes in stomatal aperture in the *KAT1::ZmnpNADP-ME* transgenic lines (Fig. 3c). Elevating the CO_2_ concentration from 400 to 700 ppm resulted in a decrease in daytime stomatal aperture in WT plants (Fig. 3d). Further increases in the CO_2_ concentration to 1200 ppm led to an additional decline in the stomatal aperture in WT plants (Fig. 3e). In contrast, stomatal size was not affected in the *KAT1::ZmnpNADP-ME* transgenic lines, neither at 700 ppm nor at 1200 ppm in comparison to the size at 400 ppm (Fig. 3).

**Figure 4:**
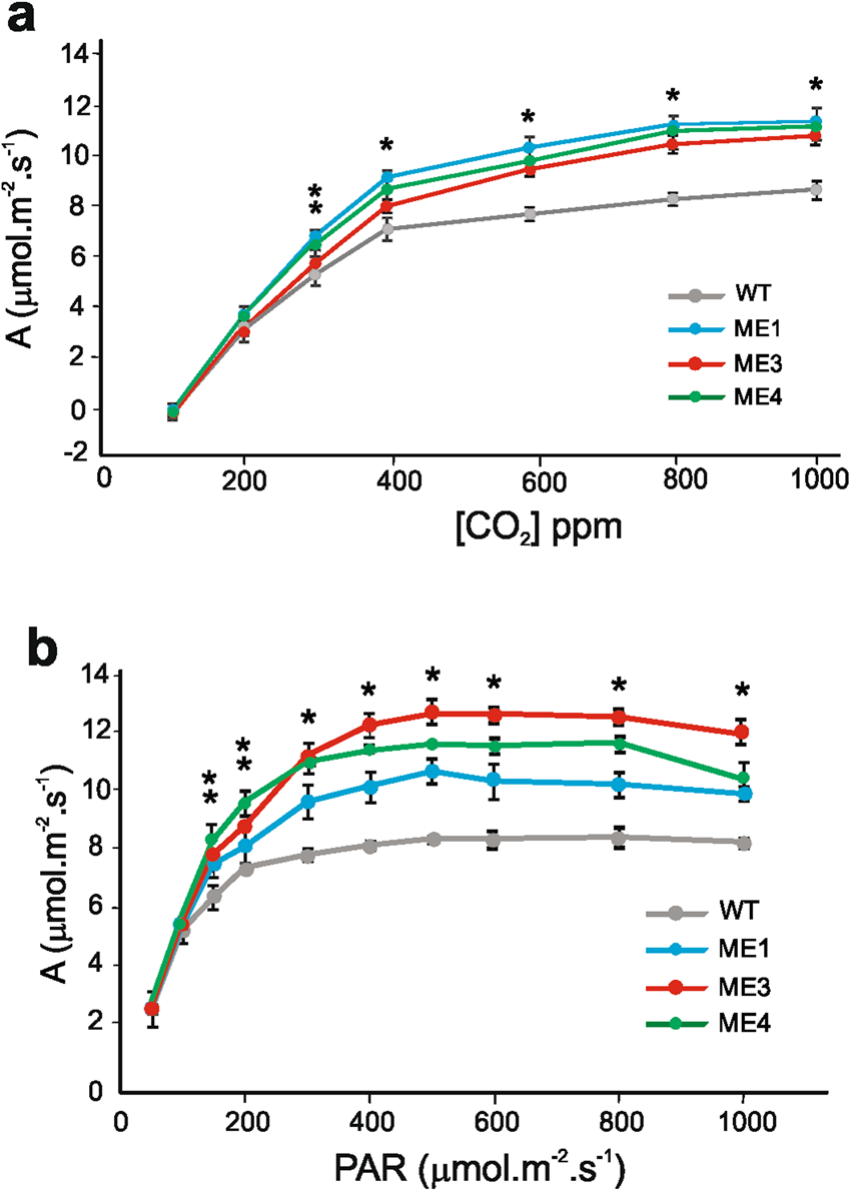
Net CO_2_ fixation rates in *KAT1::ZmnpNADP-ME* plants. CO_2_ fixation rate (A) of the third fully expanded leaves of 7 week-old WT and ME1, ME3, and ME4 as a function of CO_2_ concentration (**a**) and light intensity (**b**). The values represent the mean of five to nine independent measurements per line, using at least three different plants. * indicates that parameters measured in ME1, ME3 and ME4 are significantly different from the WT (*P* < 0.05). ** indicates that parameters measured in two of the transgenic lines are significantly different from the WT (*P* < 0.05).

As changes in stomatal behavior might influence photosynthetic efficiency, we measured the net CO_2_ uptake (A) in fully expanded leaves of transgenic and WT plants at different CO_2_ levels and PAR intensities. Significant higher net CO_2_ fixation rate (A) was found in the three independent transgenic lines at CO_2_ levels higher than 400 ppm and a light intensity higher than 300 μmol quanta m^−2^ s^−1^ of PAR (Fig. 4a,b). Similar results were obtained when net CO_2_ fixation rate (A) was graphed as a function of the calculated intercellular CO_2_ concentration (Ci, Supplemental Fig. S1). Thus, closing of stomatal pores (Fig. 3a) did not produce a negative impact on net CO_2_ uptake in the *KAT1::ZmnpNADP-ME* transgenic lines.

### Sugar level and export to the phloem in *KAT1::ZmnpNADP-ME* tobacco lines

**Figure 5:**
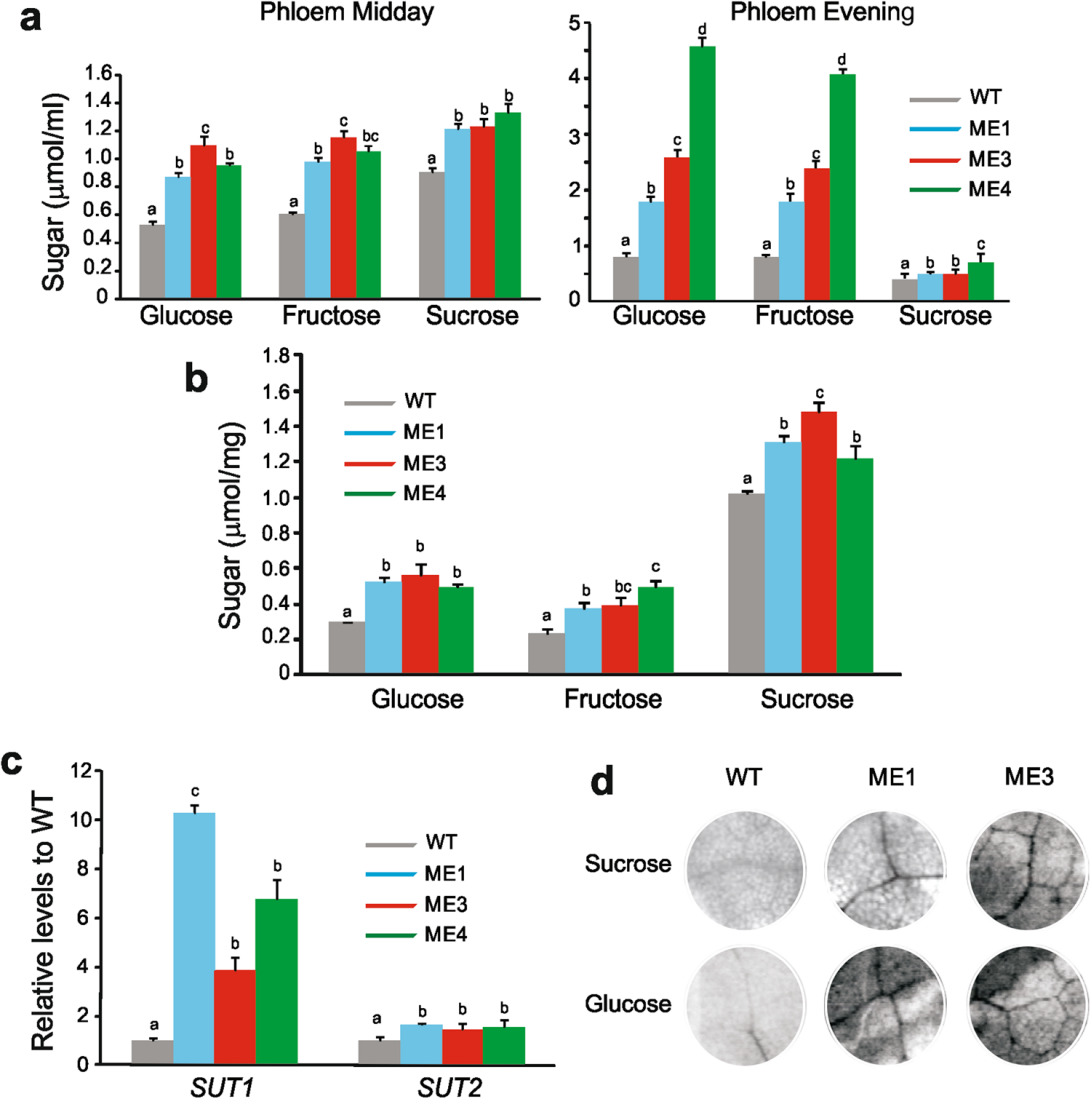
Sugar content and mobilization in *KAT1::ZmnpNADP-ME* plants. (**a**) Sucrose, glucose and fructose in phloem exudates (μmol per mL of phloem sap) at midday and the evening of 4-5 week-old WT and ME1, ME3, and ME4. Values not labeled with an identical letter are statistically significantly different (*P* < 0.05). (**b**) Sucrose, glucose and fructose in veins (μmol per mg of veins) of 4-5 week-old WT and ME1, ME3, and ME4. Values not labeled with an identical letter are statistically significantly different (*P* < 0.05). (**c**) Levels of *SUT1* and *SUT2* transcripts in leaves of ME1, ME3, and ME4 relative to WT. The values represent the mean of three independent measurements per line. Values not labeled with an identical letter are statistically significantly different (*P* < 0.05). (**d**) Sugar uptake. Autoradiographs of leaf discs of WT, ME1 and ME3 from abraded tissue and floated on either [^14^C]sucrose or [^14^C]glucose for 1 h. The intensity of the radiolabeled veins is proportional to sugar uptake.

We investigated whether the expression of ZmnpNADP-ME affected the sugar levels in phloem exudates and veins of the transgenic plants. For this, we analyzed the WT plants and transgenic lines at 4–5 weeks of growth, when all plants were at the vegetative stage (Table 2). At midday, higher sucrose, glucose, and fructose levels (from 1.5- to 2.1-fold) were found in phloem exudates of the transgenic plants compared to WT (Fig. 5a). More pronounced differences in the levels of phloem sugars (up to 6-fold increase in glucose) were found at the end of the light period (Fig. 5a). The content of sucrose, glucose and fructose were also higher in the veins of the transgenic lines compared to WT (between 1.2- to 2.1-fold higher; Fig. 5b).

As the sugar levels in phloem exudates and veins were higher in the transgenic plants than in WT, putative modifications in the transcript levels of the sucrose transporters SUT1 and SUT2 in the transgenic lines were explored. SUT1 and SUT2 are localized in the plasma membrane of companion cells and/or sieve elements^36,37^. Significantly higher transcript levels of *SUT1* (between 4 to 10-fold) and *SUT2* (between 1.4 to 1.6 fold) were found in the leaves of the transgenic lines with respect to the WT (Fig. 5c).

Considering that tobacco is a typical apoplastic sugar phloem loader^38^ we further analyzed the amount of sugar exported to the veins in discs from abraded leaf tissue exposed to ^14^C-radiolabeled sucrose or glucose. The results obtained showed that both, sucrose and glucose, were highly concentrated in the veins of the transgenic plants, indicating higher rates of sugar uptake in the veins of these plants than in the WT (Fig. 5d).

### Quantitative differential proteomic profiling of *KAT1::ZmnpNADP-ME* lines and WT

To obtain clues about putative altered pathways operating in the transgenic plants in relation to the modified phenotypic traits observed, a quantitative comparative proteomic analysis was conducted using the WT and the three transgenic lines (M1, M3 and M4). In total, 772 proteins were identified in the protein samples (Supplementary Table S1), from which 26 proteins were differently expressed when comparing the three transgenic lines *versus* WT (Table 3). From these differentially expressed proteins, 21 were increased and 5 decreased in ME1, ME3 and ME4 in comparison to the WT (Table 3).

**Table 3:** Quantitative differential proteomic analysis of *KAT1::ZmnpNADP-ME* lines and WT.

Accession (FC)	Name	GO Biological/Molecular Function
**Increased in ME1**, **ME3 and ME4 with respect to WT**		
P27154*	Phosphoenolpyruvate carboxylase	Carbon fixation
E5LCN1*	ACC oxidase 2 isoform B	Oxidoreductase activity
Q9LLS6*	60 S ribosomal protein L2 (Fragment)	Translation
Q6TKR0*	Ribosomal protein L3A	Translation
Q40597*	Tobacco W38/1 PR-1 pathogenesis-related protein	Unknown
Q43797*	Inorganic pyrophosphatase	Proton transport
Q9SDW6*	FtsZ-like protein 2	Microtubule-based process
Q9LW96*	Inositol-3-phosphate synthase	Inositol biosynthetic process
A0A075F2H1*	CONSTANS interacting protein	Peptidyl-prolyl cis-trans isomerase activity
W8S2M6*	UDP-sulfoquinovose synthase	Catalytic activity/Coenzyme binding
W8SVJ4*	Indole-3-glycerol phosphate synthase	Tryptophan metabolic process
H9CCI2*	Acyl-carrier-protein S-malonyltransferase	Metabolic process
Q0WX55*	Putative quinolinate phosphoribosyltransferase	NAD biosynthetic process
Q45KF8*	Fatty acid hydroperoxide lyase	Oxidoreductase activity
P29060*	Acidic endochitinase	Chitin catabolic process
O82030*	Histidinol-phosphate aminotransferase, chloroplastic	Histidine biosynthetic process
P93342 (5.5 ± 1.9)	14-3-3-like protein A	Protein domain specific binding
A0A068JCD7 (2.6 ± 0.4)	Fructokinase 2	Carbohydrate metabolic process
Q1W2L8 (5.0 ± 1.8)	Glutamate-cysteine ligase, chloroplastic	Glutathione biosynthetic process
Q56S59 (19.3 ± 2.5)	Phylloplanin	Defense response
Q42967 (5.4 ± 2.9)	Uroporphyrinogen decarboxylase, chloroplastic	Chlorophyll biosynthetic process
**Decreased in ME1**, **ME3 and ME4 with respect to WT**		
X5CS07*	Pyruvate kinase	Kinase activity
O82161*	Phi-1 protein	Unknown
A0A0A8JBT3 (0.25 ± 0.03)	Alpha-L-Arabinofuranosidase/beta-D-Xylopyranosidase (Fragment)	Carbohydrate metabolic process
Q9XIV8 (0.21 ± 0.08)	Peroxidase N1	Response to oxidative stress
A0A076L2F1 (0.4 ± 0.09)	Remorin 1	Unknown

Total proteins of 7 week old-leaves of WT and transgenic lines ME1, ME3 and M4 were extracted at midday and subjected to quantitative differential analysis. From the 772 proteins identified (Supplementary Table S1), 26 proteins were differentially expressed when comparing ME1, ME3, and ME4 *versus* WT. Proteins with consistently different levels when comparing the three transgenic lines and WT are shown; either from undetectable levels (indicated with *) or with fold changes higher than 2.0, *p* < *0*.*05*. In these cases, the average fold change (FC) of each protein in ME1, ME3, and ME4 with respect to WT is indicated between brackets.

The data obtained revealed that 4 proteins with altered levels in the transgenic lines are related to synthesis and/or signalling of different phytohormones. Indole-3-glycerol phosphate synthase, which is increased in transgenic lines (Table 3), is involved in indole-3-acetic acid synthesis. This phytohormone regulates many biological processes, from cell division to flowering^39^. Structurally related genes of the Phi-1 protein, increased in WT (Table 3), participate as negative regulators of cell division control in Arabidopsis and are regulated by auxin and cytokinins^40^. Other proteins related to phytohormones include ACC oxidase 2, involved in ethylene biosynthesis, and an auxin-repressed protein of unknown function.

Regarding proteins involved in flowering and life cycle time, a CONSTANS interacting protein is increased in the transgenic lines comparing to WT (Table 3). CONSTANS is a well-known regulator of flowering, and key for the transition from vegetative to reproductive stage^41^. FtsZ protein, increased in the transgenic lines (Table 3), is a key cytoskeletal component of the chloroplast division machinery and has arisen from cyanobacterial ancestors involved in cell division^42^.

Regarding changes in proteins involved in carbon metabolism, Phosphoenolpyruvate carboxylase (PEPC) was increased in the three transgenic lines in relation to WT (Table 3). PEPC is involved in carbon fixation in C_4_ and CAM plants, where it acts in coordination with NAD(P)-MEs. In C_4_ and CAM plants, higher levels of PEPC and NAD(P)-ME than C_3_ plants are usually expressed. The higher content of PEPC in the *KAT1::ZmnpNADP-ME* transgenic lines opens the question about a co-regulation of PEPC and NADP-ME as the introduction of maize NADP-ME in tobacco leads to higher levels of PEPC (Table 3). Besides, the increase in PEPC content in the three *KAT1::ZmnpNADP-ME* transgenic lines takes place along with a decrease in Pyruvate kinase content (PK, Table 3). The increase in PEPC, concomitantly with a decrease in PK would allow a derivation of PEP flux to C_4_ acid synthesis in the transgenic lines, which would provide the substrate for NADP-ME activity.

## Discussion

### Stomatal closure in *KAT1::ZmnpNADP-ME* transgenic plants: biochemical basis and impact on water usage

**Figure 6:**
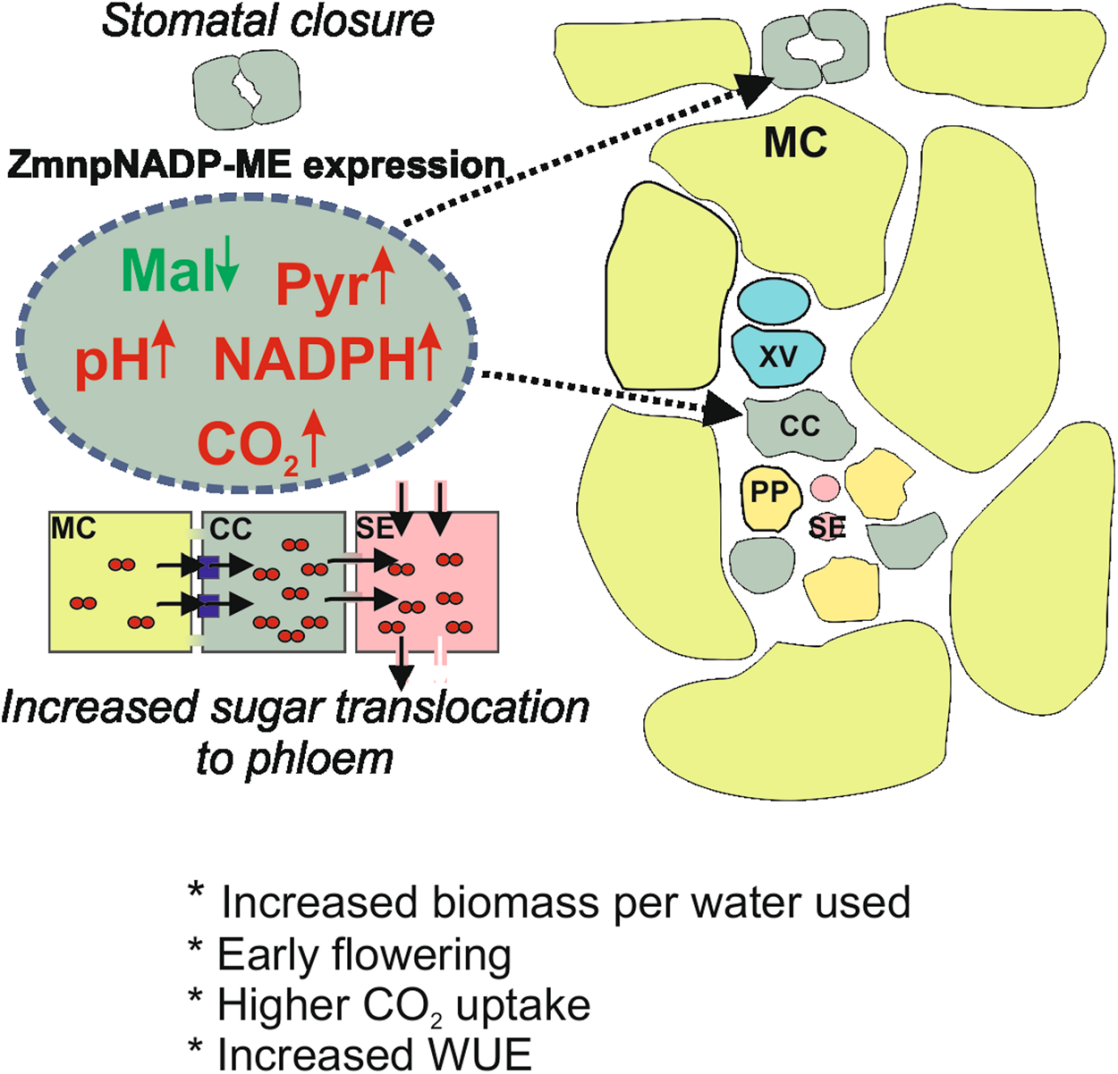
Proposed working model for *KAT1::ZmnpNADP-ME* transgenic tobacco lines. ZmnpNADP-ME, which catalyses the irreversible oxidative decarboxylation of malate to pyruvate and CO_2_, concomitant with NADP reduction, was expressed in guard cells and vascular tissue. NADP-ME activity may alter malate, pyruvate, NADPH and/or CO_2_ levels in these cells, along with possible pH modification. These changes produce stomatal closure and increased sugar export to the phloem, which are linked to the modified water usage and increased biomass production per water used. MC: mesophyll cells; CC: companion cells; SE: sieve elements; XV: xylem vessels; Mal: malate; Pyr: pyruvate.

The maize ME introduced in tobacco catalyses the irreversible oxidative decarboxylation of malate, producing pyruvate, CO_2_ and NADPH and it is not subjected to inhibition by malate as its C_4_ photosynthetic counterpart^30,31,43^. The transgenic lines express a functional and active ME in guard cells as accounted by *in situ* activity assays (Fig. 1b and c). Thus, the activity of ZmnpNADP-ME may lead to a decrease in malate, concomitant with an increase in CO_2_, pyruvate and NADPH levels, specifically in guard cells (Fig. 6). *KAT1::ZmnpNADP-ME* transgenic plants show reduced stomatal pore sizes with respect to WT under normal growth conditions (400 ppm CO_2_ and 200 μmol quanta m^−2^ s^−1^ of PAR), both at the beginning and at the end of the light period (Fig. 3a,b). Several different experimental approaches have shown the key role of malate in regulating stomatal size by altering turgor pressure, so the decrease in malate levels due to NADP-ME activity in guard cells may be directly involved in the closure of stomata in transgenic lines. Moreover, it has been proposed that malate is metabolized to starch or transported from guard cells to the apoplastic space during stomatal closure^14^. Furthermore, the possible decrease in malate levels may also induce a decrease of stomatal opening, considering the role of malate as osmolyte in guard cells. Besides, a decrease of malate may also affect chloride levels, as it has been shown that malate activates chloride channels and plays a key role as a signaling molecule in the regulation of anion fluxes in guard cells^22^.

On the other hand, elevated intracellular CO_2_ levels in guard cells due to NADP-ME activity may lead to activation of guard cell plasma-membrane anion channel currents, as CO_2_ has been described as a major signal regulator of stomatal movements^44,45^. The finding that incubation of epidermal strips under high CO_2_ levels induces stomatal closure in WT, but not in transgenic lines (Fig. 3d,e), is in line with the speculation that CO_2_ signaling, due to high CO_2_ levels, may be already activated in these lines. In this sense, although Ci levels were not significantly modified in transgenic lines with respect to WT (Supplementary Fig. S1), it may be possible that elevated CO_2_ levels may take place only in guard cells.

Besides, modification of pyruvate levels in guard cells may also be involved in stomatal aperture alteration in transgenic lines, as it has been shown that a mitochondrial pyruvate carrier mediates ABA regulation of guard cell ion channels^46^. In addition, acidification of epidermal strips does not induce the opening of stomata in the transgenic lines, as it does in the WT (Fig. 3c); thus, this lack of response to pH indicates that the biochemical status of the guard cells of transgenic lines superimposes the response to pH, avoiding stomatal opening.

Alternatively, the lower stomatal aperture in the transgenic lines may also be due the higher content of sugars in the phloem (Fig. 5); as it has been shown that apoplastic accumulation of sugars can induce stomatal closure in a mechanism dependent of photosynthesis and mediated by the transpiration stream^47^.

Overall, the biochemical modifications produced by ME activity in guard cells may act synergistically to induce stomatal closure in *KAT1::ZmnpNADP-ME* transgenic lines (Fig. 6). In turn, stomatal closure of *KAT1::ZmnpNADP-ME* transgenic lines impacts positively on water consumption per day, which was significantly reduced with respect to that in the WT (Table 2). Considering that the total life cycle of the transgenic lines is substantially reduced compared with WT (Table 2), the impact on water savings by the transgenic plants analyzed in this work is extremely high when compared to WT plants.

### Increased sugar export in *KAT1::ZmnpNADP-ME* transgenic plants superimposes to the negative impact that stomatal closure may exert on photosynthesis

Although stomatal closure is expected to produce a decrease in photosynthesis due to lower CO_2_ levels in mesophyll cells, this is not the case for *KAT1::ZmnpNADP-ME* transgenic plants. On the contrary, CO_2_ uptake is increased in the transgenic lines in comparison to WT at CO_2_ levels higher than 400 ppm (Fig. 4a) and light intensity higher than 300 μmol quanta m^−2^ s^−1^ of PAR (Fig. 4b).

Because higher NADP-ME activity was detected in the vascular tissue of the transgenic lines (Fig. 1b,d), we wondered if sugar translocation to phloem would be altered in *KAT1::ZmnpNADP-ME* transgenic lines. We found that higher NADP-ME activity in the vascular tissues of transgenic lines (Fig. 1b,d) is accompanied by an increase in sugar export from the mesophyll cells to the veins (Fig. 5d). This increased sugar export is traduced in higher sucrose, glucose and fructose levels in phloem exudates in transgenic lines, both at midday and evening (Fig. 5a). Consistently, sugar level is increased in veins of transgenic lines in comparison to WT (Fig. 5b). All measurements concerning sugar level and export (Fig. 5) were performed at the vegetative stage of the plants (Table 2), so the differences between transgenic lines and WT are not due to a higher sink demand.

Phloem loading of sugars is mediated by sucrose transporters (SUT) located in companion cells, which mediate a H^+^/sucrose co-transporter^37,48^. Sucrose transporters are tightly regulated at various levels, and their modification has a great impact on plant growth and photosynthesis^49–51^. It has been shown that the activity of SUT1 is highly modulated at post-translational level, being regulated by changes in quaternary structure, redox state, and by the pH status^52,53^. Thus, biochemical modifications mediated by NADP-ME activity in vascular-associated cells, such as lower malate and changes of pH and NADPH/NADP ratio (Fig. 6), may affect the activity of sucrose transporters, leading to an enhanced export of sugars to the phloem (Fig. 5). Moreover, we also found higher levels of *SUT1* and *SUT2* in transgenic lines in relation to WT (Fig. 5c), which also supports the increase in sugar export (Fig. 5d). Considering that transcriptional regulation of phloem sucrose transporters is mediated by sugar levels^54^; higher activity of sucrose transporters and hence, translocation of sugars to the phloem, may in turn be related to higher levels of *SUT1* and 2 in the transgenic lines (Fig. 5c). The increase in fructose and glucose in phloem exudates and veins in transgenic lines (Fig. 5a,b) may be explained by sucrose cleavage catalyzed by apoplasmic invertases, although activation of hexoses transporters may also take place in transgenic lines (Fig. 5d)^55^.

It is well known that an excess of carbon metabolites in source organs triggers the negative feedback regulation of photosynthesis^56^. Because of this, phloem loading has been pointed out as a critical target to increase crop yields; it is expected that higher sucrose removal from the mesophyll, especially under conditions of low sink demand, would avoid sugar accumulation in the leaf mesophyll cells and thus, CO_2_ uptake rates would be expected to remain high^57^. The results obtained in our work are in line with this assumption, as higher export of sugars (Fig. 5) is correlated with increased CO_2_ uptake (Fig. 4) although stomata are more closed than in WT (Fig. 3).

Sugars not only serve as energy and carbon skeleton, they also fulfil a wide range of regulatory functions in plants and would function as signalling molecules coordinating the expression of many genes^58^. The modification of the level of several different proteins in transgenic lines in comparison to WT (Table 3) is in line with the role of sugars as signalling molecules regulating growth and development in plants. Besides, sucrose is a well-known signal leading to flowering^59^. We propose that increased sugar levels in the phloem (Fig. 5) may also mediate the decrease in flowering time of the transgenic lines and the decrease of their life cycle (Table 2). Moreover, the function of several proteins, which levels are altered in transgenic lines, would be related to early flowering and decrease life cycle (Tables 2 and 3).

### Malic enzyme expression in particular cell compartments: A biotechnology tool for enhanced WUE and productivity in an enriched CO_2_ world

Previous work on the constitutive expression of the photosynthetic ZmNADP-ME in tobacco plants caused decreased stomatal conductance and compromised plant development^15^. Here, we found that the restricted expression of ZmnpNADP-ME in particular cell compartments is traduced in an opposite phenotype (Fig. 6). This highlights the great impact that modification of particular compartments, like guard cells and companion cells of phloem, exert on the whole plant physiology.

The predicted increase in the atmospheric concentration of CO_2_ is expected to have a deep effect on our ecosystem and would negatively impact on overall stomatal conductance, by reducing both stomatal apertures and the total numbers of stomata per unit leaf area^44^. It is postulated that this global effect would be beneficial for limiting water loss; however, it would have a potential cost on photosynthesis^44^. The results obtained in our work, suggest that *KAT1::ZmnpNADP-ME* lines would not have any negative feedback on photosynthetic activity under high CO_2_ levels; on the contrary, in these plants photosynthetic performance is significantly higher than in WT at elevated CO_2_ (Fig. 4a). Growing the transgenic lines in elevated [CO_2_] would better test their performance in such environment. In addition, it would prove their potential for enhancing phloem loading and alleviating negative feedback on photosynthesis. Overall, our results suggest that in a future world with elevated CO_2_ the enhancement of sugar export capacity and of carbon utilization are important issues to maximize photosynthesis and yield.

In summary, we developed an approach to avoid plant water loss by expressing a maize NADP-ME isoform in tobacco guard cells and vascular tissue. The mechanism behind this effect is a more pronounced stomatal closure in the transgenic plants than in the WT. Due to an increased rate of sugar export to phloem this mechanism is associated with enhanced carbon fixation. Minimization of water loss by stomatal closure without a penalty in carbon assimilation rate is a great challenge in crop improvement. The strategy presented in this work also accelerates plant reproductive development, which is also a favorable trait for crops cultivated primarily for the use of their seeds.

## Methods

### Plasmid Construct and Plant Transformation

The full-length sequence of the plastidic *ZmnpNADP-ME* cDNA (Gene Bank: AY315822) and a 1.8 kbp promoter region of the *Arabidopsis thaliana* Potassium channel 1 (KAT1, Gene Bank: U25088; At5g46240) were cloned between the EcoRI and XbaI restriction sites of a modified version of the pGreen II vector bearing the hygromicin resistance gene^24^. *Agrobacterium tumefaciens* (GV3101) containing the “helper” pSOUP plasmid was transformed with the *pGreenII::KAT1::ZmnpNADP-ME* construct by the freeze thaw method. *Nicotiana tabacum KAT1::ZmnpNADP-ME* transgenic lines were generated by *Agrobacterium*-mediated transformation. Leaf discs were incubated for 30 min with *A*. *tumefasciens* carrying the plasmid *pGreenII::KAT1::ZmnpNADP-ME* and co-cultivated for three days in the dark in Murashige and Skoog medium containing 2.7 g L^−1^ Phytagel, supplemented with 1 mg L^−1^ 6-benzylaminopurine and 0.1 mg L^−1^ 1-naftalenacetic acid. The explants were kept in the same medium supplemented with 250 mg L^−1^ cefotaxime and 40 mg L^−1^ hygromycin at 25 °C, 16:12 h photoperiod and 70 μmol m^−2^ s^−1^ active photon flux density. Shoots were rooted and hygromycin-resistant primary transformants were transferred into soil and grown under 25 °C, 12:12 h photoperiod and 200 μmol quanta m^−2^ s^−1^ of PAR. Homozygous lines were obtained after several rounds of selection.

### Plant growth conditions

*N*. *tabacum* (L. cv. Petit Havana SR1) wild-type (WT) and transgenic plants (ME1, ME3, and ME4) were grown from seeds in a compost:sand:perlite mixture (2:1:1 by volume). Seedlings were transferred to a greenhouse with a 30/18 °C 12/12 h day/night period and 200 μmol quanta m^−2^ s^−1^ of PAR and ambient CO_2_ concentration (400 ppm). Tobacco plants were irrigated at 90% field capacity (FC) conditions by weighing the individual pot of each plant every day^60^.

### Phenotype analyses

After 10 weeks of growth, plant height, internode length and number of leaves of WT and KAT1::ZmnpNADP-ME plants were recorded for at least five plants belonging to each type. The maximum length of the leaves of WT (leaves 1 to 23 from base to top) and transgenic lines (leaves 1 to 16 from base to top) were measured. Flowering time was recorded as the time of growth of each plant until setting the first flower buds. Life cycle time was recorded as the time of growth until producing mature seeds, immediately after which the plants began the senescence process.

### Water consumption determination and biomass production

After 4 weeks of growth, tobacco WT and *KAT1:ZmnpNADP-ME* lines were transferred to weighted 5 L pots without drainage holes. One plant per pot was harvest. The pots were weighted every day and water was added to each pot to assure 90% Field Capacity. As control, three pots were kept without plants in order to discount the amount of water evaporated from the soil. Pots containing WT and transgenic lines were randomly distributed in the same greenhouse. Water added per day and consumed until completing 11 weeks of growth was recorded. After 11 weeks of growth, plants were harvested and aerial (leaves and stems) and terrestrial (roots) dry weight (DW) were measured using at least 3 plants of each line and two different sets of plants.

### Native PAGE

Abaxial epidermal tissue from tobacco leaves was manually peeled^61^. Minor veins were retained in the resulting epidermal strips (sample called leaf peelings plus veins). The peels were immediately frozen in liquid nitrogen. Total soluble protein from the leaf peelings plus veins and stems was extracted and desalted as previously described^34^ and used for NADP-ME activity measurement. Ten mU of NADP-ME were loaded on native PAGE and assayed for NADP-ME activity as in^25^. Protein concentration was determined following the method of Bradford^62^.

### *In situ* NADP-ME activity assay

*In situ* NADP-ME activity assay was carried out in 14 days-old old tobacco seedlings as described in Gerrard Wheeler *et al*.^35^. Alternatively, staining for enzyme activity was carried out in leaf epidermis peelings. For this purpose, abaxial leaf peels were manually extracted and fixed with 2% (w/v) paraformaldehyde and 1 mM DTT in PBS Buffer (137 mM NaCl; 2.7 mM KCl; 10 mM Na_2_HPO_4_; 2 mM KH_2_PO_4_, pH 7.4) during 1 h at 4 °C and then rinsed overnight in water at 4 °C. Staining for NADP-ME activity was carried out as described by^35^. The tissue was mounted with 50% (v/v) glycerin and visualized using a Zeiss Axiover 25 (Spectra) microscope.

### Determination of stomatal aperture

Leaves of 7-week-old *N*. *tabacum* plants were bleached with 96% (v/v) ethanol during 10 min at 100 °C and with 5% (w/v) NaOH in 50% (v/v) ethanol during 5 min at 100 °C. This treatment ensures that the cells are fixed and allows stomatal pore size measurements. After extensive washes with abundant water, the samples were incubated in a 50% (w/v) NaClO solution until discoloration and washed. The tissue was stained with saturated Safranin O and mounted with 0.15% (w/v) gelatin in 50% (v/v) glycerin. Guard cells were observed with a LabPhot-2 Nikon microscope. Pore sizes were measured in more than 100 stomata using at least six independent preparations.

Modulation of stomatal aperture by acidification was carried out incubating leaf epidermis under 200 μmol m^−2^ s^−1^ PPDF for 3 h in acidic buffer (50 mM KCl; 10 mM butyrate, pH 5.8). The “clamping” cytosolic pH achieved with this buffer is 6.0. The guard cell response to high CO_2_ was done incubating leaf peelings in the light during 15 min in a chamber with moderate or high levels of CO_2_ (700 and 1200 ppm, respectively).

### Gas exchange analysis

Net CO_2_ fixation rates (A; μmol CO_2 _m^−2^ s^−1^) were determined using a LI-6400XT equipment (LI-COR). The third fully expanded leaves of 7-week-old *N*. *tabacum* plants were used at midday; 6 h after lights were turn on. Different CO_2_ concentrations (between 50 to 800 ppm CO_2_) at constant PAR (200 μmol quanta m^−2^ s^−1^), as well as different PAR intensities (between 25 to 1,000 μmol quanta m^−2^ s^−1^) at constant CO_2_ concentration (400 ppm) were used. All of the photosynthetic measurements were taken at a constant air flow rate of 400 μmol s^−1^ and 26 ± 2 °C. Five to nine measurements were made for each plant, using the third fully expanded leaf. At least three different plants were used for both the WT and the transgenic plants (ME1, ME3 and ME4).

### RT-PCR

Total RNA was isolated from 0.04–0.10 g of tissue using the Trizol method (Invitrogen). The integrity of the RNA was verified by agarose electrophoresis. The quantity and purity of RNA were determined. First-strand cDNA was synthesized with MoMLV-reverse transcriptase following the manufacturer’s instructions (Promega, Madison, WI, USA) and using 2 µg of RNA and oligo(dT).

### Quantitative real-time PCR

Relative expression was determined by performing quantitative real-time PCR (QRT-PCR) in an iCycler iQ detection system and the Optical System Software version 3.0a (Bio-Rad, Hercules, CA, USA), using the intercalation dye SYBRGreen I (Invitrogen), as previously described^34^. PCR controls, cycling parameters and melting curves were carried out as reported elsewhere^34^. Relative gene expression was calculated using the Comparative 2^— ΔΔ*C*T^ method and elongation factor 1α (*ef1α*) as reference gene. The following oligonucleotide primers were used: *ZmnpNADP-ME*for (5′-CCAAATGGCTTCCTTCAATG-3′) and *ZmnpNADP-M*Erev (5′-CCGAATCCAGGGAAAATG-3′); *SUT1*for (5′-CCTTGACTCTCTTTGCTGTCCT-3′) and *SUT1*rev (5′-CTGCAACTGCTCCAACAATAAA-3′); and *SUT2*for (5′-AGAGAAGGTGCATTTGGTTTG-3′) and *SUT2*rev (5′-AGCAAGAGGAATGCCAAGAAG-3′). Each RNA sample was run in triplicate and repeated in at least three independent set of treatments.

### Determination of sugar content

Phloem samples were obtained by making small punctures with a hypodermic needle into petioles of 4 to 5-week-old *N*. *tabacum* plants at midday (6 h after lights were turn on) and at the evening (10 h after the lights were turn on). The first exuding droplet was discarded and the subsequent exudate was collected and immediately frozen in liquid nitrogen for further analysis. Minor veins were manually cut using a razor blade from 4 to 5-week-old *N*. *tabacum* plants at midday, weighed, and frozen in liquid nitrogen for further analysis. Sugar extraction, liquid partition and concentration to dryness were performed as previously described^63^. The dried residue was re-dissolved in distilled water and the content of glucose, fructose and sucrose was determined enzymatically according to Stitt *et al*.^64^. The amount of sugars is expressed in mmol/mL of phloem exudate and µmol/mg of fresh vein weigh.

### Sugar vein loading

Vein loading with sugars was determined as previously described^38^. Leaves were abraded and the cuticle was removed using a razor blade. Discs were cut under distilled water with a cork borer and floated, abraded side down, on 2 mL solution containing 20 mM Mes-NaOH buffer, pH 5.5, 2 mM CaCl_2_, and 1 mM of either [^14^C]sucrose or [^14^C]glucose (40 kBq mL^−1^) for 1 h at room temperature. The discs were then rinsed 3 times for 20 min each, flash-frozen with dry ice, freeze-dried, pressed flat, and exposed to Kodak BioMax MR film for 24–48 h.

### Quantitative differential proteomic profiling

Protein extraction from 7-week-old *N*. *tabacum* leaves (at midday) was performed by using 50 mM Tris-HCl pH 8.0, 5.0 mM EDTA, 0.1% (v/v) Triton X-100, and protein inhibitor cocktail (Sigma). Two different pools, composed each one by the third leaf from three different plants of each type (WT, ME1, ME3 and ME4), were processed as biological replicates. Crude protein extracts were cleared by centrifugation and quantified by Bradford assay^62^. One hundred microgram of each protein crude extract was precipitated by trichloroacetic acid (TCA; 1/5 100% (p/v)). Pelleted proteins were dissolved in 50 µL 8 M urea; 10 mM DTT at 56 °C for 45 min. Denatured proteins were then reduced with 10 mM DTT and treated with 20 mM iodoacetamide to prevent disulfide bonds reformation and precipitated by trichloroacetic acid (TCA; 1/5 100% (p/v)). Protein digestion and Mass Spectrometry analysis were performed at the Proteomics Core Facility CEQUIBIEM, at the University of Buenos Aires/CONICET (National Research Council) as follows: samples were resuspended in 50 mM ammonium bicarbonate pH 8 and digested overnight with sequencing-grade modified trypsin (Promega) and then cleaned with Zip-Tip C18 (Merck Millipore) to extract the salts. Desalted Peptides were analyzed by nanoHPLC (EASY-nLC 1000, ThermoScientific, Germany) coupled to a mass spectrometer with Orbitrap technology (Q-Exactive with High Collision Dissociation cell and Orbitrap analyzer, ThermoScientific, Germany). Peptide Ionization was performed by electrospray. The data obtained were analyzed using the Proteome Discoverer 2.1 software (ThermoScientific, Germany) for identification and relative quantitation using the area of each protein; and Perseus software (Max Planck Institute of Biochemistry)^65^ was used for statistical analysis. Accession number in UniprotKB database (http://www.uniprot.org/uniprot/); score, coverage and peptides used for identification; area in each biological repetition; as well as other parameters from of each protein are listed in Supplementary Table S1. Differential expressed proteins (26 proteins) comparing WT with the three in transgenic lines (ME1, ME3 and ME4) are listed in Table 3. Proteins with different levels in only one biological replication; or in one, or even two, transgenic lines in comparison to the biological replications of WT were not considered as differently expressed.

### Statistical analysis

Data were tested using one-way analysis of variance (ANOVA). Minimum significance differences were calculated by the Holm-Sidak test (α = 0.05) using the Sigma Stat Package.

## Electronic supplementary material

Supplementary Figures

Supplementary Table
