# Supplementary material for: Improved water use efficiency and shorter life cycle of Nicotiana tabacum due to modification of guard and vascular companion cells
Source: Sci Rep. 2018 Mar 12;8:4380. doi: 10.1038/s41598-018-22431-5 (PMC5847574; doi:10.1038/s41598-018-22431-5)

Improved water use efficiency and shorter life cycle of *Nicotiana tabacum* due to modification of guard and vascular companion cells

Muller GL, Lara MV, Oitaven P, Andreo CS, Maurino VG, & Drincovich MF

Supplementary Fig. S1. a. CO_2 fixation rate (A) of the third fully expanded leaves of 7 week-old WT and ME1, ME3, and ME4 as a function of intracellular CO_2 concentration (Ci). * indicates that parameters measured in ME1, ME3 and ME4 are significantly different from the WT ($P < 0.05$). **b.** Intracellular CO_2 concentration (Ci). as a funcion of atmospheric CO_2 concentration (Ca).

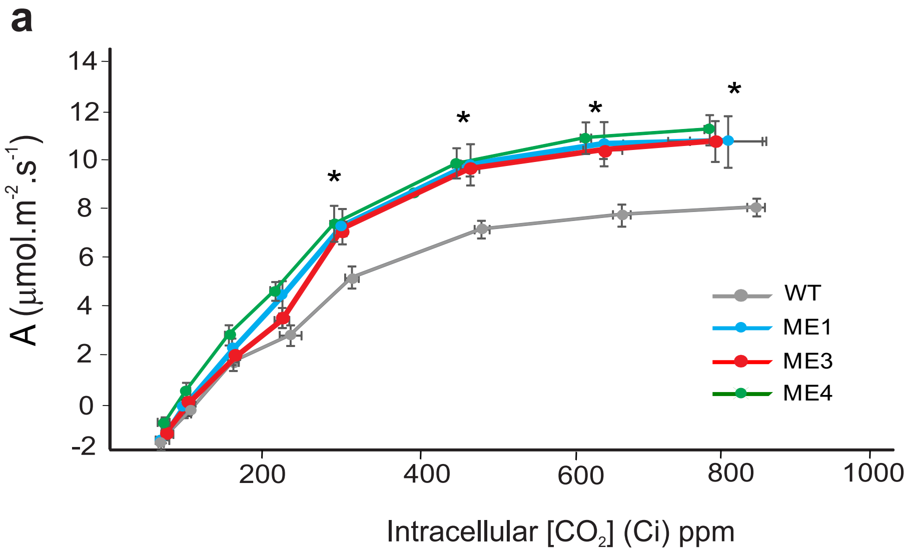

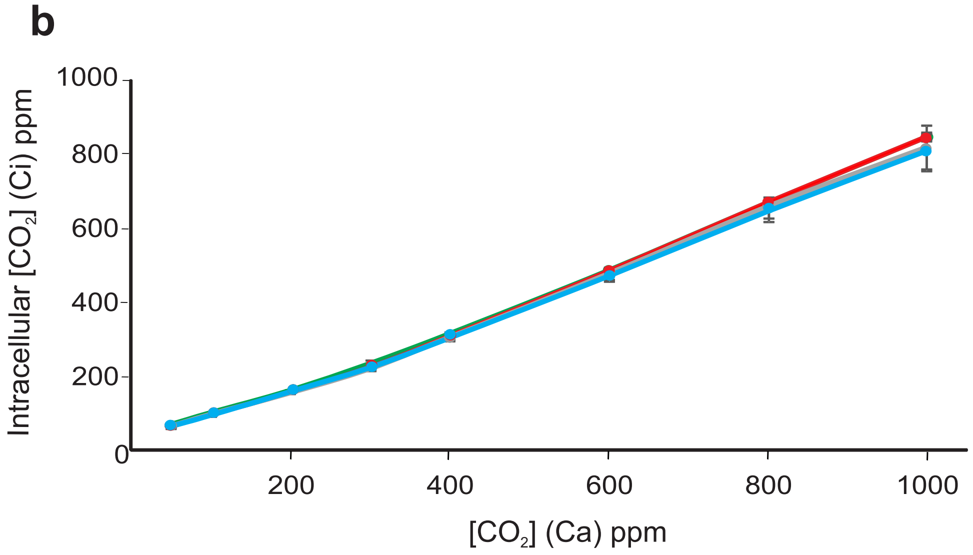

Improved water use efficiency and shorter life cycle of *Nicotiana tabacum* due to modification of guard and vascular companion cells
Muller GL, Lara MV, Oitaven P, Andreo CS, Maurino VG, & Drincovich MF
Supplementary Fig. S2. Full-length gel shown in Figure 1b

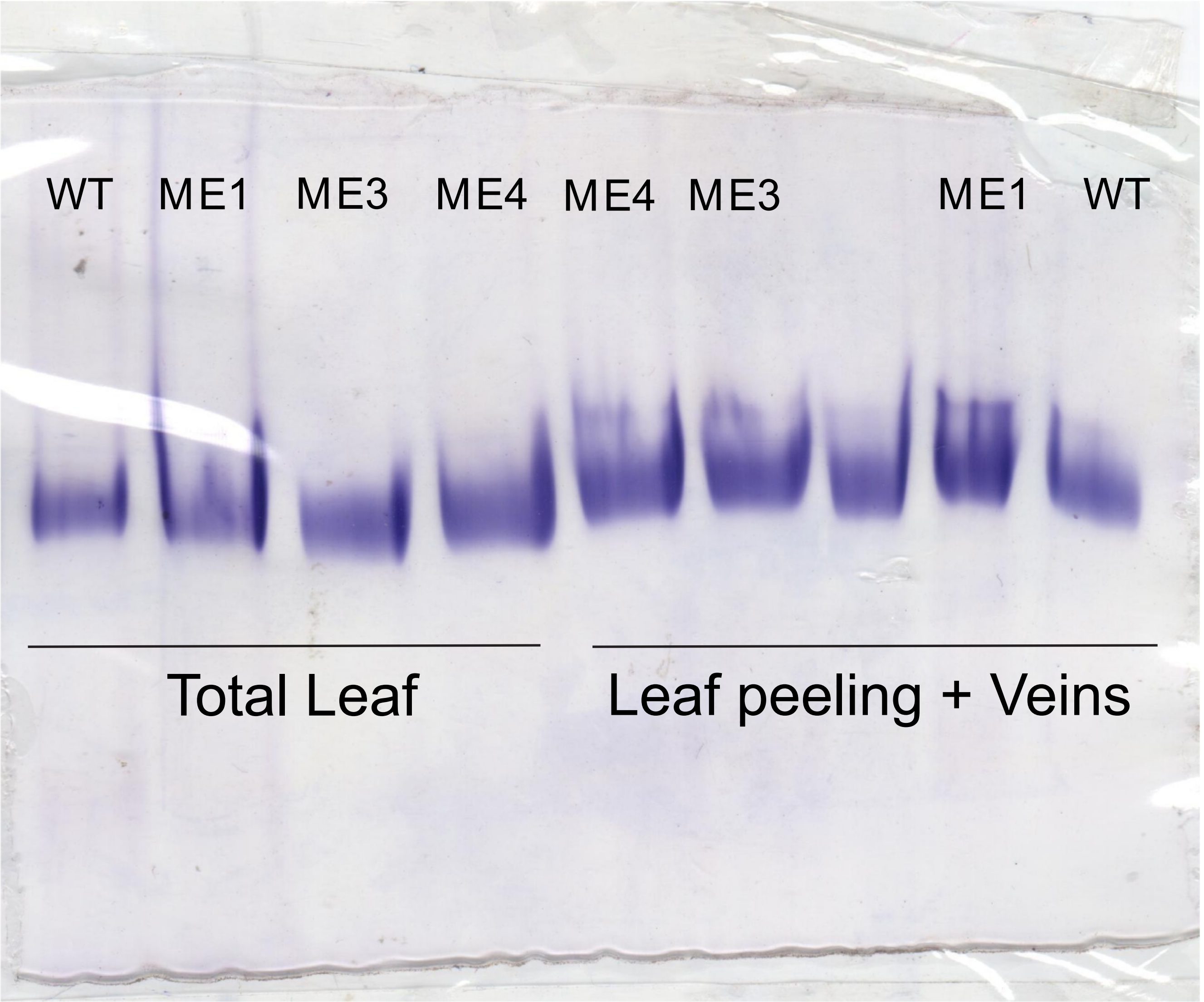

Supplement: Supplementary file 1 — Supplementary Figures [file 41598_2018_22431_MOESM1_ESM.pdf]
